# SENP3 sensitizes macrophages to ferroptosis via de-SUMOylation of FSP1

**DOI:** 10.1016/j.redox.2024.103267

**Published:** 2024-07-14

**Authors:** Xuelian Chen, Jizhuang Wang, Peilang Yang, Hsin-Ying Liu, Shan Zhong, Chenghao Lu, Min Gao, Dan Liu, Jie Zhang, Jiaqiang Wang, Shan Ma, Wenao Wang, Hanting Zhu, Xiong Zhang, Yan Liu

**Affiliations:** Department of Burn, Ruijin Hospital, Shanghai Burn Institute, Shanghai Jiao Tong University School of Medicine, Shanghai, China

**Keywords:** SENP3, Ferroptosis, FSP1, Macrophage

## Abstract

Ferroptosis, driven by an imbalance in redox homeostasis, has recently been identified to regulate macrophage function and inflammatory responses. SENP3 is a redox-sensitive de-SUMOylation protease that plays an important role in macrophage function. However, doubt remains on whether SENP3 and SUMOylation regulate macrophage ferroptosis. For the first time, the results of our study suggest that SENP3 sensitizes macrophages to RSL3-induced ferroptosis. We showed that SENP3 promotes the ferroptosis of M2 macrophages to decrease M2 macrophage proportion in vivo. Mechanistically, we identified the ferroptosis repressor FSP1 as a substrate for SUMOylation and confirmed that SUMOylation takes place mainly at its K162 site. We found that SENP3 sensitizes macrophages to ferroptosis by interacting with and de-SUMOylating FSP1 at the K162 site. In summary, our study describes a novel type of posttranslational modification for FSP1 and advances our knowledge of the biological functions of SENP3 and SUMOylation in macrophage ferroptosis.

## Highlights

• SENP3 sensitizes ferroptosis of M2 macrophages.
• SENP3 highly expressed in diabetic wounds.
• SENP3 reverses the ferroptosis repressing effect of FSP1 by de-SUMOylating FSP1 at the K162 Site.

## Graphical abstract

Table of Contents Ferroptosis and the SUMO protease SENP3 regulate macrophage function, but whether SENP3 regulates macrophage ferroptosis remains unclear. Chen et al. showed that SENP3 can sensitize macrophages to ferroptosis by interacting with and de-SUMOylating FSP1, which can delay diabetic wound healing by reducing the proportion of M2 macrophages.

**Figure:**
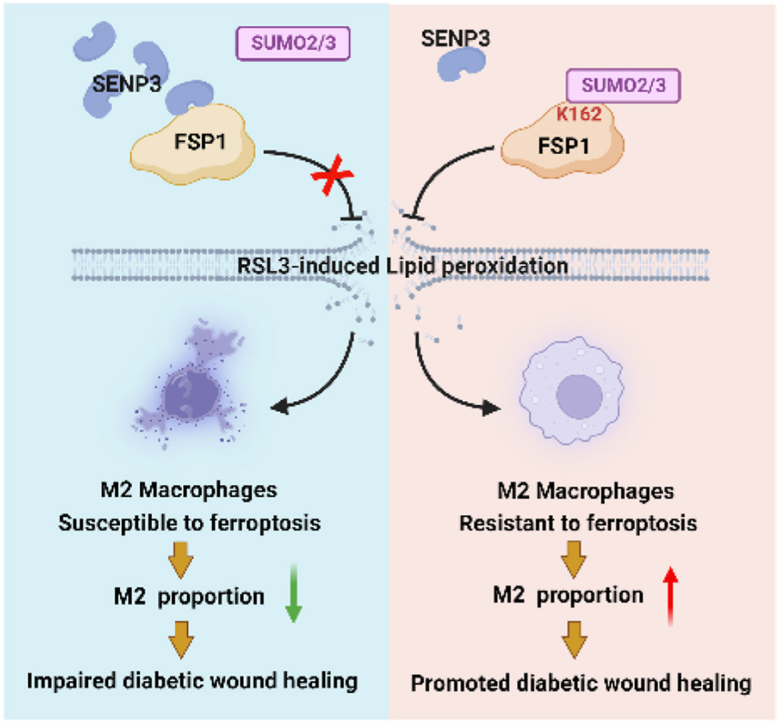

## 1 Introduction

Ferroptosis has recently been identified as an iron-dependent programmed cell death that is driven by lipid peroxidation, with mechanisms distinct from those of apoptosis, necrosis, and pyroptosis [[1], [2], [3]]. Initiated by an imbalance in redox homeostasis, ferroptosis is regulated by iron metabolism and diverse cellular metabolism involving amino acids, glucose, and lipids [4,5]. Recently, many studies have investigated the regulatory role that ferroptosis plays in macrophage function and inflammatory response [3]. Evidence suggests that damage-associated molecular patterns and alarmins are released during ferroptosis of macrophages, amplifying inflammation and tissue damage [6,7]. In addition, a recent study indicated that macrophages in different polarization states exhibit diverse susceptibilities to ferroptosis. Activated (M1) macrophages mediate inflammation, which is vital for microbial killing [8]. Alternatively activated (M2) macrophages limit inflammatory response and are responsible for tissue regeneration [9]. Previous studies suggested that M1 macrophages show significantly increased tolerance to ferroptosis compared to M2 macrophages, leading to a higher M1/M2 ratio under conditions of oxidative stress [10]. The pathologically increased M1/M2 ratio of macrophages is a major contributor to multiple diseases, including chronic non-healing wounds. However, the underlying molecular mechanisms of ferroptosis require further elucidation.

Recently, there is growing recognition of the significance of protein post-translational modifications (PTMs) in ferroptosis [11,12]. Studies have shown that ferroptosis regulators can undergo PTMs, such as phosphorylation, acetylation, glycosylation, ubiquitination, and succination, which play indispensable roles in regulating ferroptosis [13,14]. It is important to determine whether there are new novel PTMs involved in regulating ferroptosis. SUMOylation, a recently discovered type of PTM that generally refers to SUMO1 and SUMO2/3 modification, is possibly related to ferroptosis [14,15]. The level of protein SUMOylation is regulated by both SUMOylation and de-SUMOylation and is mediated by the SUMO-specific protease SENP [16]. SENP3 is the predominant oxidative stress-sensitive protein in the SENP family [17]. It is unclear whether SUMOylation regulates ferroptosis. In addition, the role of the de-SUMOylation enzyme SENP3 in regulating ferroptosis remains unclear.

Glutathione peroxidase 4 (GPX4) is considered as the key repressor of ferroptosis, catalyzing phospholipid hydroperoxides reduction [18]. Inactivating GPX4 with RSL3 or conditional GPX4 knockout can induce ferroptosis in mouse embryonic fibroblasts [2,19]. The Conrad team and the Olzmann team recently identified ferroptosis suppressor protein 1 (FSP1) as the next major ferroptosis suppressor in addition to GPX4, by using a genetic suppressor screen or synthetic lethal CRISPR-Cas9 screen [20,21]. Studies have suggested that FSP1 exerts ferroptosis inhibition parallel to GPX4 activity by reducing coenzyme Q10 and vitamin K [22,23]. It remains largely unknown whether SUMOylation regulates the function of FSP1. In addition, it remains unclear what role SENP3 plays in regulating FSP1.

This study is the first to explore the relationship between SENP3 and macrophage ferroptosis. We clarified that SENP3 regulates macrophage phenotype by controlling ferroptosis to influence diabetic wound healing. Mechanistically, we clarified that SENP3 regulates macrophage ferroptosis through SUMOylation of FSP1. Moreover, we identified K162 as the predominant SUMOylation site for FSP1. Overall, this study advances our knowledge of the biological roles of SENP3 and SUMOylation during ferroptosis and chronic non-healing wounds.

## 2 Methods

### 2.1 Animals

Animals were obtained from the Shanghai Laboratory Animal Center. C57BL/6 mice at 5–12 weeks of age were used, as indicated in the figures and legends. The animals were housed under specific pathogen-free (SPF) conditions. Senp3 flox/flox (SENP3^fl/fl^) or Senp3 flox/flox; Lyz2-Cre (SENP3^cko^) mice were established as previously described [24]. The Institutional Animal Care & Use Committee of Shanghai Jiao Tong University School of Medicine approved the protocol (RJ2023021).

### 2.2 Cell culture and cell transfection

Cells were obtained from the American Type Culture Collection. The RAW264.7 cells were maintained using RPMI 1640 medium (Gibco). HEK293T and HT1080 cells were maintained using DMEM medium (Gibco). The cells were cultured with medium containing 10 % fetal bovine serum (Gibco) and 1 % penicillin/streptomycin (NCM Biotech), 5 % CO_2_ at 37 °C. In HEK293T cells, plasmid DNA and shRNAs were transfected using PEI (Yeasen).

### 2.3 Bone marrow-derived monocytes (BMDMs) isolation and culture

BMDMs from SENP3^fl/fl^ or SENP^cko^ mice were isolated as described previously [24]. Mice were sacrificed and subsequently sterilized. The cell suspensions were obtained by flushing the bones using RIPM 1640 (Gibco) and then filtered by 70-μm cell strainers. The cells were maintained in a medium supplemented with 25 ng/ml M-CSF (Abclonal) for 7 days to differentiate.

### 2.4 Cell viability assays

We seeded 2 × 10^4^ BMDMs, 1 × 10^4^ RAW 264.7, or 1 × 10^4^ HT1080 cells on 96-well plates overnight. Then, we incubated cells with ferrostatin-1 (MCE), viFSP1 (MCE), LPS (100 ng/ml) and IL-4 (25 ng/ml) for 24 h. Then, cell viability was tested 5 h after RSL3 treatment using a Cell Counting Kit (Beyotime). The cell viability was expressed as a relative value of the control sample.

### 2.5 LDH release assays

We seeded 2 × 10^4^ BMDMs, 1 × 10^4^ RAW 264.7, or 1 × 10^4^ HT1080 cells on 96-well plates overnight. Then, we incubated cells with ferrostatin-1 (MCE), viFSP1 (MCE), LPS (100 ng/ml), and IL-4 (25 ng/ml) during seeding on 96-well plates. Next, cells were treated with RSL3 (Selleck). The cell death rate was determined by the LDH detection kit (Promega) following the instructions. Briefly, cell lysates were prepared by using 10x lysis from the kit as a lysate sample as total LDH release. 50 μL culture supernatant was obtained as the medium sample. Lysate and medium samples were each mixed with reagents for 30 min, following which the addition of a 50 μL stop solution initiated a measurement by a microplate reader at 490 nm. Cell death levels were quantified as a relative value of total LDH release.

### 2.6 BODIPY 581/591 C11 staining

We seeded BMDMs or HT1080 (1 × 10^6^ cells per well) on 6-well dishes overnight. Cells were treated with RSL3 for 1 h and incubated with 2.5 μM BODIPY 581/591 C11 (Thermo Fisher), resuspended in 300–500 μL of Hanks' balanced salt solution (HBSS, Gibco). Subsequently, a flow cytometer (CytoFLEX Beckman Coulter) was used to analyze. Data were analyzed using CytExpert.

### 2.7 SYTOX Green

BMDMs or HT1080 were seeded on 6-well dishes (1 × 10^6^ cells per well) overnight. Cells were incubated with 1 μM RSL3 for 5 h 1:20000 SYTOX Green (Thermo Fisher) was added and incubated for 10 min, then images were taken by fluorescence microscopy randomly at least 5 images for each sample. Data were analyzed by Image J and results were expressed as dead cells/total cells%.

### 2.8 Zymosan peritonitis RSL3-ferroptosis model

SENP3^fl/fl^ and SENP3^cko^ mice were administered an intraperitoneal injection of zymosan A (Sigma-Aldrich, from Saccharomyces cerevisiae) at a dosage of 100 mg/kg as previously described [10]. Seventy-two hours after zymosan administration, the mice were injected with saline or RSL3 (40 mg/kg) intraperitoneally. Five hours after the RSL3 administration, peritoneal exudates were isolated.

### 2.9 Flow cytometry

Macrophages were derived from mouse peritoneal exudates as previously described [10] and stained at 4 °C for 30 min using fluorescence-conjugated antibodies: Brilliant Violet 510™ anti-mouse CD45 (1:200, 30-F11 clone, 103138, Biolegend); Alexa Fluor® 700 anti-mouse/human CD11b (1:200, M1/70 clone, 101222, Biolegend); Brilliant Violet 785™ anti-mouse F4/80 (1:200, BM8 clone, 123141, Biolegend); FITC anti-mouse CD80 Antibody (1:200, 16-10A1 clone, 104705, Biolegend); PE anti-mouse CD206 Antibody (1:200, C068C2 clone, 141705, Biolegend); APC750 Live/dead (1:100). All assays were performed by CytoFLEX Beckman Coulter. Data were analyzed using CytExpert.

### 2.10 Mice and rat diabetic model

For mice diabetic model, 5 weeks mice (C57BL/6wild-type mice, SENP3^fl/fl^, and SENP3^cko^ were administered with streptozotocin (dissolved in citrate buffer; Yeasen) with 80 mg/kg and 40 mg/kg a week later. For the rat diabetic model, 6-week-old rats were intraperitoneally injected with 60 mg/kg and 30 mg/kg a week later with streptozotocin. Blood glucose and weight were monitored weekly. The mice with blood glucose levels above 16.7 mM were fed for 6–8 weeks and used for the following experiments. Citrate buffer alone injected mice or rats were used as no diabetic controls.

### 2.11 Full-thickness diabetic wounds model

Full-thickness skin wounds were carried out as previously described [11]. On days 3, 5, 7, 11, and 15 post-surgery, these wounds were documented by photography, and the extent of wound closure was quantified. The image analysis was conducted using ImageJ. On days 5, 7, and 15 post-surgery, the wounds with 5 mm surrounding tissues were analyzed.

### 2.12 Histology, immunohistochemistry, and immunofluorescence of skin wounds

The sections of skin wounds were stained through H&E and Masson staining. Macrophages, and angiogenesis in the skin wounds, paraffin-embedded mouse or rat wound sections were stained by antibodies against SENP3 (1:400; Cell Signaling Technology, 5591), F4/80 (1:100; Invitrogen, MA1-91124), CD68 (1:400; Abcam, ab955), AGR-1 (1:400; Cell Signaling Technology, 93668), iNOS (1:400, Invitrogen, PA1-036) and CD31 (1:1000, Abcam, ab281583). For immunohistochemistry, sections were stained with a DAB Substrate kit (Vector Laboratories, SK-4100) following secondary antibodies. The Digital Pathology Slide Scanner from Shanghai Runnerbio Technology CO., Ltd was used to scan the slides. For immunofluorescence, sections were stained using fluorescence-labeled secondary antibodies (1:400, Invitrogen; A11006 or A32766 or A10042) for 60 min. Following staining of the nuclei using DAPI (Yeasen), the slides were captured using an Olympus digital camera or scanned by a digital slide scanner 3DHISTECH. All images were analyzed by Image J software.

### 2.13 FerroOrange staining

We seeded BMDMs (1 × 10^6^ cells per well) on a 3.5 cm fluorescent imaging dish. Cells were stimulated with 1 μM RSL3 for 4 h. Following three washes in HBSS, 1 μM FerroOrange (DOJINDO, F374) was added to the cells. Then, we randomly took at least five images for each sample using a Zeiss LSM880. Data were analyzed using Image J, with results expressed as fluorescence intensity.

### 2.14 Glutathione peroxidase (GPX) activity assay

Intracellular GPX activity was quantified by coupling to NADPH oxidation in the presence of excess glutathione reductase using a cellular GPX assay kit (Beyotime, S0056) as previously described [25]. Briefly, BMDMs were cultured in 10 cm dishes and treated with 1 μM RSL3 for 2 h. After that, the supernatant of cell lysates was collected to perform the GPX activity assay following the instructions. The protein level of each sample was determined by a BCA assay (Pierce, Thermo Scientific). GPX activity was calculated and expressed as mU/mg protein.

### 2.15 Sh-RNA, plasmid, and mutagenesis

sh-NC and sh-SENP3, described in Supplementary Information Table 1, were constructed as described previously [24]. The plasmids were constructed and used as described previously [24,26]. The plasmids including human FSP1, mouse FSP1, and human SENP3 were generated by Shanghai Xitu Biotechnology Co., Ltd. The coding sequence region of human SENP3 (NM_015670.6) was synthesized (de novo synthesis) and cloned to the pLVX-EF1a-IRES-puro vector. The coding sequence region of human FSP1 (NM_001198696.2) with N-terminal 3 × Flag was synthesized (de novo synthesis) and cloned between *Xho*I and *Bam*HI sites of the pLVML-CMV-IRES-puro vector. The coding sequence region of mouse FSP1 (NM_001039194.3) with C-terminal 3 × Flag was synthesized (de novo synthesis) and cloned between *Xba*I and *Bam*HI sites of the pLVX-EF1a-IRES-puro vector. The human HA-FSP1 was generated in our laboratory based on the WT plasmid using pCDH lentivectors. The human FSP1 mutants, including the 3 × flag-FSP1 K43R mutant, the 3 × flag-FSP1 K162R mutant, and the 3 × flag-FSP1 K225R mutant, were generated in our laboratory based on the WT plasmid using the Vazyme 2 × Phanta Flash Master Mix (P510). The mouse FSP1–K162R mutant expression plasmid was also generated by Shanghai Xitu Biotechnology Co., Ltd., that is based on the WT plasmid by site-directed mutagenesis. The primers for mutagenesis are shown in Table 1. The plasmid details are listed in Table 2.

### 2.16 Preparation of lentiviral particles, stable expression, or knockdown by transfection

A lentiviral packaging system comprising a transfer plasmid, pMD2.G, and psPAX2 was co-infected into HEK293T cell. Following a 48-h incubation, cell culture supernatant was filtered through 0.45-μm syringe filters to transduce the cell line of interest.

We seeded the cells in a 10 cm dish one day before transfection. Supernatants containing viral particles were added for 12 h and then were changed. Two days after the transfection, cells were subjected to the selection process involving puromycin (0.5 μg/ml for HT1080, 2 μg/ml for RAW 264.7) to obtain cells with stable expression SENP3, FSP1, or FSP1 K162R in HT1080 and RAW 264.7 cells. To achieve stable expression, the cells were maintained under selective conditions.

For stable knockdown SENP3 by sh-NC and sh-SENP3, RAW 264.7 cells were used for the following experiment 48 h after infection.

### 2.17 Immunoblotting (IB)

We carried out the IB as described [27]. The antibodies that were utilized in the experiment were: SENP3 (Cell Signaling, 5591), SUMO2/3 (Cell Signaling, 4971), SUMO1 (Cell Signaling, 4940), RH (QIAGEN, 34610), HA (Cell Signaling, 3724), Flag (Sigma, F3165) and β-actin (Cell Signaling, 4970), vinculin (Proteintech, 66305), tubulin (Proteintech,66362), GFP (Proteintech, 66002), FSP1 (Cell Signaling, 24972), FSP1 (Proteintech, 68049-1).

### 2.18 Flag immunoprecipitation assay

The flag immunoprecipitation assay was performed using the routine protocol as previously described [26]. After transfection, the cells were lysed using a lysis buffer followed by centrifugation at 13000*g* for 15 min. Then, Anti-Flag M2 Magnetic beads (Sigma Aldrich, M8823) were added to cell lysates to incubate overnight at 4 °C. Next, we analyzed the eluted samples through IB.

### 2.19 Ni-NTA pull-down assay

The Ni-NTA pull-down assay was carried out using the previously described method [26]. Briefly, we transfected cells with RH-tagged plasmids and then lysed them following the manufacturer's protocol. Subsequently, the cell lysates were incubated with HisSep Ni-NTA MagBeads (Yeasen) for 2 h. Next, we analyzed the eluted RH-tagged proteins through IB.

### 2.20 Statistical analysis

An unpaired *t*-test or multiple unpaired t-tests were used to compare differences between the two groups. Either one-way analysis of variance (ANOVA) or two-way ANOVA was used to analyze group differences. Statistical significance is defined as a *p*-value less than 0.05. The results are represented as means ± sd. The statistical analysis for this study was conducted using GraphPad Prism 9.0.

## 3 Results

### 3.1 SENP3 sensitizes macrophages to RSL3-induced ferroptosis

**Fig. 1:**
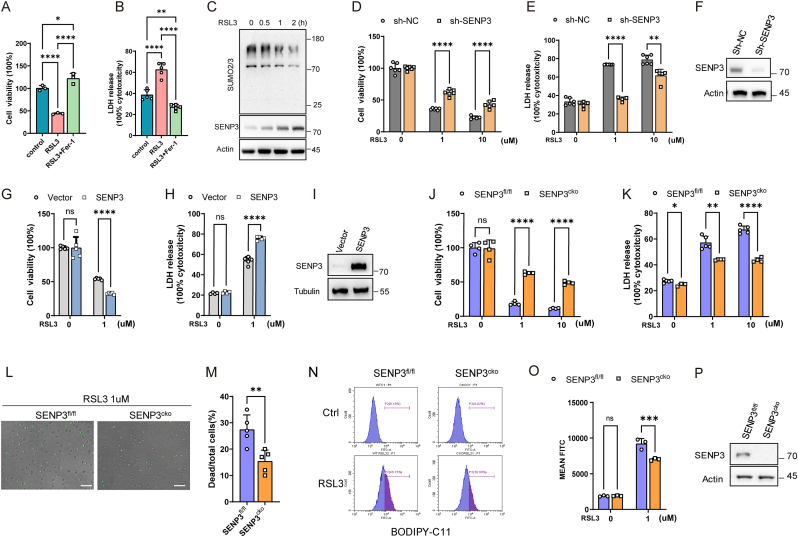
SENP3 sensitizes macrophages to RSL3-induced ferroptosis. **(A**–**B)** RAW 264.7 macrophages were incubated with vehicle, RSL3 (1 μM), and RSL3 (1 μM) + Fer-1 (1 μM) for 5 h. Then, the viability (A) and the Lactate dehydrogenase (LDH) release (B) of RAW 264.7 macrophages were tested. **(C)** RAW 264.7 macrophages were stimulated with RSL3 (1 μM) for the indicated time, and SENP3 and SUMO2/3 modification were measured by IB. **(D**–**F)** RAW 264.7 macrophages stable knockdown of SENP3 or not were constructed by sh-SENP3 and sh-NC. These cells were then incubated with vehicle, RSL3 (1 μM), and RSL3 (10 μM) for 5 h. Next, the viability (D) and Lactate dehydrogenase (LDH) release (E) of RAW 264.7 macrophages were tested. SENP3 expression in sh-NC and sh-SENP3 RAW 264.7 macrophages were validated by IB (F). **(G**–**I)** RAW 264.7 macrophages were stably overexpressing SENP3 or empty vector as control. SENP3 overexpressing and control RAW 264.7 macrophages were then incubated with vehicle or RSL3 (1 μM) for 5 h. Next, the viability (G) and LDH release (H) of these cells were tested. Western blots validated SENP3 expression in control and SENP3 RAW 264.7 macrophages (I). **(J**–**M)** Naïve bone marrow derived macrophages (BMDM) from SENP3^fl/fl^ and SENP3^cko^ (SENP3^Lyz2−/-^ mice) were treated with vehicle, RSL3 (1 μM), and RSL3 (10 μM) for 5 h. Then, the viability (J) and LDH release (K) of these cells were tested. SYTOX Green staining of SENP3^fl/fl^ and SENP3^cko^ BMDMs (L) and statistics analysis of dead/total cells% were shown (M). **(N–P)** SENP3^fl/fl^ and SENP3^cko^ BMDMs were incubated with RSL3 (1 μM) for 1 h. Lipid peroxidation was evaluated by BODIPY 581/591 C11 staining (N) and statistics analysis of mean FITC (O). Western blots validated the expression of SENP3 in SENP3^fl/fl^ and SENP3^cko^ BMDMs (P). Scale bars, 100 μm. Data are shown as means ± SD. Data are representative of three independent experiments. ****p < 0.0001, **p < 0.01, *p < 0.05, One-way ANOVA (A, B). ****p < 0.0001, ***p < 0.001, **p < 0.01, *p < 0.05, Multiple unpaired t-tests (D, E, G, H, J, K, O). **p < 0.01, unpaired *t*-test (M).

To elucidate whether SENP3 and SUMOylation play roles in regulating the ferroptosis of macrophages, we treated RAW 264.7 macrophages with RSL3 to induce ferroptosis [10,28,29]. According to the results, RSL3 suppressed cell viability and elevated LDH release in RAW 264.7 cells, and these effects were rescued by the ferrostatin-1 (Fer1) treatment (Fig. 1A and B). These results suggested that the ferroptosis of macrophages can be induced by RSL3.

During RSL3-induced macrophage ferroptosis, SENP3 rapidly accumulated in response to RSL3 stimulation along with decreased SUMO2/3 modification (Fig. 1C), whereas SUMO1 modification remained stable (Supplementary Fig. 1A), which was further ascertained by statistical analysis (Supplementary Fig. 1B). SENPs can regulate SUMO2/3 modification [30,31]. Therefore, we attempted to determine whether the accumulation of SENP3 can lead to decreased SUMO2/3 modification during ferroptosis by stably knocking down SENP3 expression in RAW 264.7 macrophages. SENP3 knockdown significantly reversed the decrease in SUMO2/3 modification in response to RSL3 stimulation (Supplementary Figs. 1C and D). However, in untreated cells, SENP3 knockdown did not affect SUMO2/3 modification, indicating that other SENP family members may compensate for the loss of SENP3 under basal conditions. Previous studies have shown that SENP3 expression increases under oxidative stress [32]. SENP3 accumulated in RSL3-stimulated RAW264.7 cells within 120 min. Meanwhile, antioxidant N-acetyl cysteine (NAC) reversed the increase in SENP3 expression (Supplementary Figs. 1E and F). The data indicate that RSL3-induced SENP3 accumulation is ROS-dependent. Together, these data suggest that SENP3 expression increases and SUMO2/3 modification decreases during RSL3-induced macrophage ferroptosis.

Next, we determined whether SENP3 regulates RSL3-induced macrophage ferroptosis. Knockdown of SENP3 expression promoted cell viability and suppressed LDH release in response to RSL3 stimulation in RAW 264.7 cells (Fig. 1D–F). Moreover, SENP3 overexpressing RAW264.7 cells exhibited reduced cell viability and increased LDH release (Fig. 1G–I). The above results suggest that SENP3 sensitizes RAW 264.7 cells to RSL3-induced ferroptosis.

Furthermore, BMDMs from SENP3^fl/fl^ or SENP3^cko^ mice were used to validate the regulation of SENP3 on macrophage ferroptosis. SENP3^cko^ BMDMs exhibited increased cell viability and reduced LDH release compared with those from SENP3^fl/fl^ mice during RSL3-induced ferroptosis (Fig. 1J and K). Moreover, a SYTOX Green assay was performed to assess cell death. We observed that SENP3^cko^ BMDMs showed decreased positive cells than SENP3^fl/fl^ BMDMs (Fig. 1L and M). In addition, SENP3^cko^ BMDMs also exhibited relatively decreased lipid peroxidation compared with that of SENP3^fl/fl^ BMDMs after RSL3 stimulation (Fig. 1N-P). These data suggest that SENP3 sensitizes BMDMs to RSL3-induced ferroptosis. Collectively, these results demonstrated that SENP3 makes macrophages more susceptible to ferroptosis.

### 3.2 SENP3 promotes the ferroptosis of M2 macrophages

**Fig. 2:**
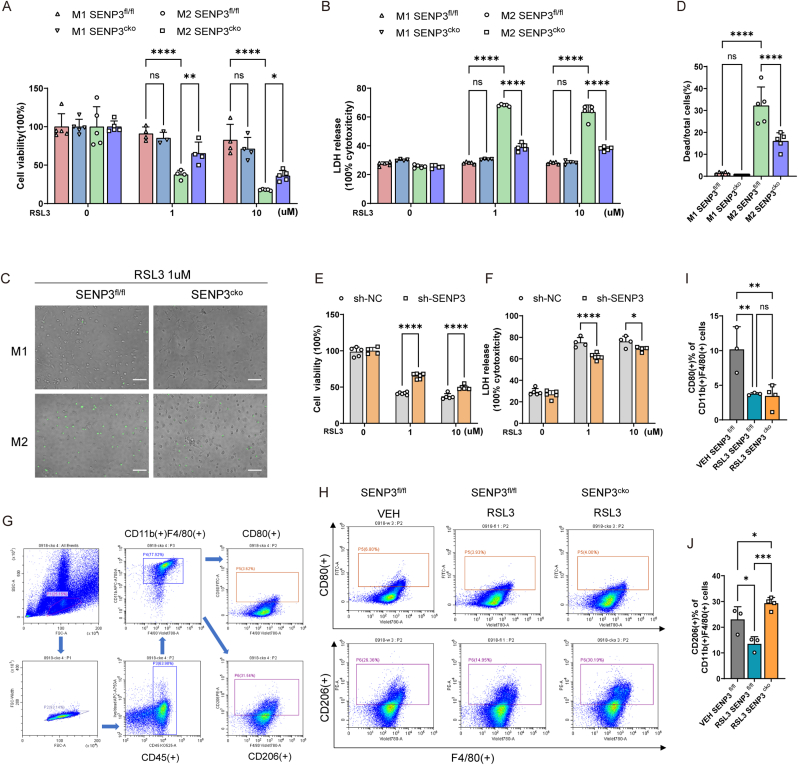
SENP3 promotes the ferroptosis of M2 macrophages. **(A**–**D)** BMDMs from SENP3^fl/fl^ and SENP3^cko^ mice were incubated with LPS (100 μg/ml; M1 macrophages) and IL-4 (25 ng/ml; M2 macrophages) for 24 h independently, followed with RSL3 for 5 h. Next, the viability (A) and LDH release (B) of M1 and M2 macrophages from SENP3^fl/fl^ and SENP3^cko^ mice were tested. SYTOX Green staining of M1 and M2 macrophages from SENP3^fl/fl^ and SENP3^cko^ mice (C) and statistics analysis of dead/total cells% were shown (D). **(E**–**F)** Sh-NC and sh-SENP3 RAW 264.7 macrophages were treated by IL-4 (25 ng/ml) for 24 h, followed by RSL3 for 5 h. Next, the viability (E) and LDH release (F) of sh-NC and sh-SENP3 M2 macrophages were tested. **(G**–**J)** Peritoneal macrophages in a mouse model of zymosan-peritonitis plus RSL3 were collected for flow cytometry test. Gating strategy of CD45^+^ CD11b + F4/80+ peritoneal macrophages (G). Representative flow cytometry images show CD206+ or CD80^+^ cells gated from CD45^+^ CD11b + F4/80+ peritoneal macrophages (H). Statistics analysis of CD45^+^ CD11b + F4/80 + CD206+ macrophages and CD45^+^ CD11b + F4/80+ CD80^+^ macrophages in each group (I, J). Scale bars, 100 μm. Data represents mean ± SD, n = 3 or 4 biologically independent samples. ****p < 0.0001, **p < 0.01, *p < 0.05, two-way ANOVA (A, B). ****p < 0.0001, *p < 0.05, Multiple unpaired t-tests (E, F). ****p < 0.0001, ***p < 0.001, **p < 0.01, *p < 0.05, One-way ANOVA (D, I, J).

Previous studies have shown that M2 macrophages were more sensitive than M1 macrophages to RSL3-induced ferroptosis [10]. Herein, we polarized SENP3^fl/fl^ and SENP3^cko^ BMDMs to M1 and M2 macrophages respectively, followed by stimulation with RSL3. The data suggested that after RSL3 stimulation, M2 macrophages showed significantly decreased cell viability and increased LDH release, while the cell viability and LDH release did not markable change in RSL3-treated M1 macrophages (Fig. 2A and B). The SYTOX Green assay results also showed fewer positive cells of M1 than M2 after RSL3 treatment (Fig. 2C and D). The results indicated that M2 macrophages were more sensitive than M1 macrophages to RSL3-induced ferroptosis. Moreover, compared with SENP3^fl/fl^ M2 macrophages, SENP3^cko^ M2 macrophages showed markedly increased cell viability and decreased LDH release after RSL3 treatment (Fig. 2A–D). We used SENP3-knockdown RAW264.7 macrophages to validate the role of SENP3 expression in the ferroptosis of M2 macrophages via IL-4 polarization. Similarly, compared with IL-4-polarized sh-NC RAW264.7 cells, IL-4-polarized SENP3-knockdown RAW264.7 cells exhibited increased cell viability and decreased LDH release (Fig. 2E and F). These results suggest that loss of SENP3 expression confers a protective effect on M2 macrophages against RSL3-induced ferroptosis.

To test whether SENP3 drives ferroptosis of macrophages in vivo, we used a mouse model of zymosan-induced sterile peritonitis followed by RSL3 injection, in which RSL3 was injected 72 h after zymosan treatment, and the peritoneal macrophages were mainly M2 macrophages [10]. SENP3^fl/fl^ and SENP3^cko^ mice were injected with zymosan followed by RSL3 injection. Next, peritoneal cells were collected to test macrophage polarization by flow cytometry, and the gating strategy used for CD45^+^ CD11b + F4/80+ peritoneal macrophages is shown (Fig. 2G). The results showed that in both the SENP3^fl/fl^ and SENP3^cko^ mice, among the CD45^+^ CD11b + F4/80+ macrophages, the percentage of CD80^+^ M1 macrophages was less than 5 %, while the percentage of CD206+ M2 macrophages was at least twice as high as that of M1 macrophages (Fig. 2H–J). More importantly, the proportion of M2 macrophages was lower in the SENP3^fl/fl^ group compared to the SENP3^cko^ group and the control group (Fig. 2H–J). Overall, SENP3 decreased the M2 macrophage proportion by enhancing the susceptibility to ferroptosis of macrophages.

### 3.3 SENP3 is highly expressed in macrophages of diabetic wounds

**Fig. 3:**
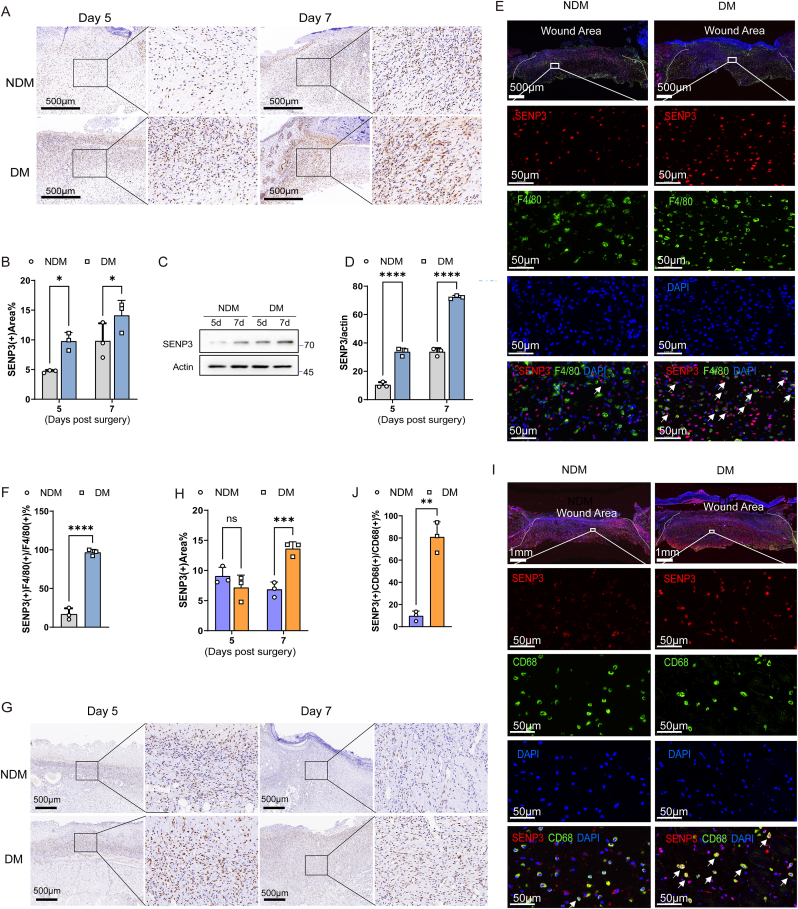
SENP3 is highly expressed in macrophages of diabetic wounds. C57BL/6 wild-type mice or SD rats were intraperitoneally injected with streptozotocin (STZ) to induce diabetics (DM), or PBS as control (NDM). Full-thickness wounds were created on these NDM or DM mice or rats and collected at the indicated time. **(A**–**F)** wounds from NDM or DM mice were collected at 5 days and 7 days post-surgery. The skin wounds were subjected to immunohistochemistry of SENP3 (A); and statistics analysis of SENP3 positive area/wound area% (B). The level of SENP3 in mice skin wounds was evaluated by IB (C); and statistics analysis of SENP3/actin (D). Wounds collected at 5 days post-surgery were subjected to immunofluorescence staining of SENP3 or F4/80 (E) and statistics analysis of F4/80(+) SENP3(+)/F4/80(+) cells% (F). **(G**–**J)** wounds from NDM or DM rats were collected at 5 days and 7 days post-surgery. The skin wounds were subjected to immunohistochemistry of SENP3(G) and statistics analysis of SENP3 positive area/wound area% (H). Immunofluorescence staining of SENP3 or CD68 of wounds collected at 5 days post-surgery (I) and statistics analysis of CD68(+) SENP3(+)/CD68(+) cells% (J). Scale bars, as indicated in the figures. Data represents mean ± SD, n = 3 biologically independent samples. ****p < 0.0001, ***p < 0.0001, *p < 0.05, two-way ANOVA (B, D, H). **p < 0.01, ****p < 0.0001, unpaired *t*-test (F, J).

The above results suggested that the SENP3 mediated decrease in the proportion of M2 macrophages proportion was ferroptosis-dependent. Next, to explore whether SENP3 functions in diabetic wounds, we first examined its expression in wounds. In normal skin, SENP3 was primarily expressed in the hair follicles and the epidermis, while increasing expression of SENP3 was observed in migrating tongues and wound area on day 3 and day 5 post-injury, followed by a decrease on day 14 (Supplementary Fig. 2A). Increased SENP3 expression was observed in the wound area at days 5 and 7 post-injury in both normal (NDM) and diabetic (DM) wounds (Fig. 3A). However, increased SENP3 expression was shown in diabetic wounds compared to normal wounds at 5 and 7 days post-injury (Fig. 3A and B). Western blotting revealed greater than 3-fold and 2-fold upregulation of SENP3 protein expression on day 5 and day 7, respectively, in diabetic wounds compared with normal wounds (Fig. 3C and D). The above results suggest that SENP3 was more highly expressed in diabetic wounds than normal wounds.

The pathologically elevated M1/M2 ratio delays the healing process in diabetic wounds [33]. Furthermore, a previous study showed that SENP3 may regulate macrophage polarization [34]. We therefore explored whether SENP3 accumulated in macrophages in diabetic wounds. Immunofluorescence assays using macrophage markers confirmed that macrophages in mouse skin wounds express SENP3 (Fig. 3E). Statistical analysis demonstrated increased SENP3 expression in macrophages of diabetic wounds compared with those in normal wounds (Fig. 3F).

Furthermore, greater SENP3 expression was also detected in rat diabetic rat wounds (Fig. 3G and H) and macrophages (Fig. 3I and J) than in normal controls. These findings indicated a potential pathological role of SENP3 in macrophages and diabetic wounds.

### 3.4 SENP3^cko^ mice exhibited an increased proportion of M2 macrophages and promoted diabetic wound healing

**Fig. 4:**
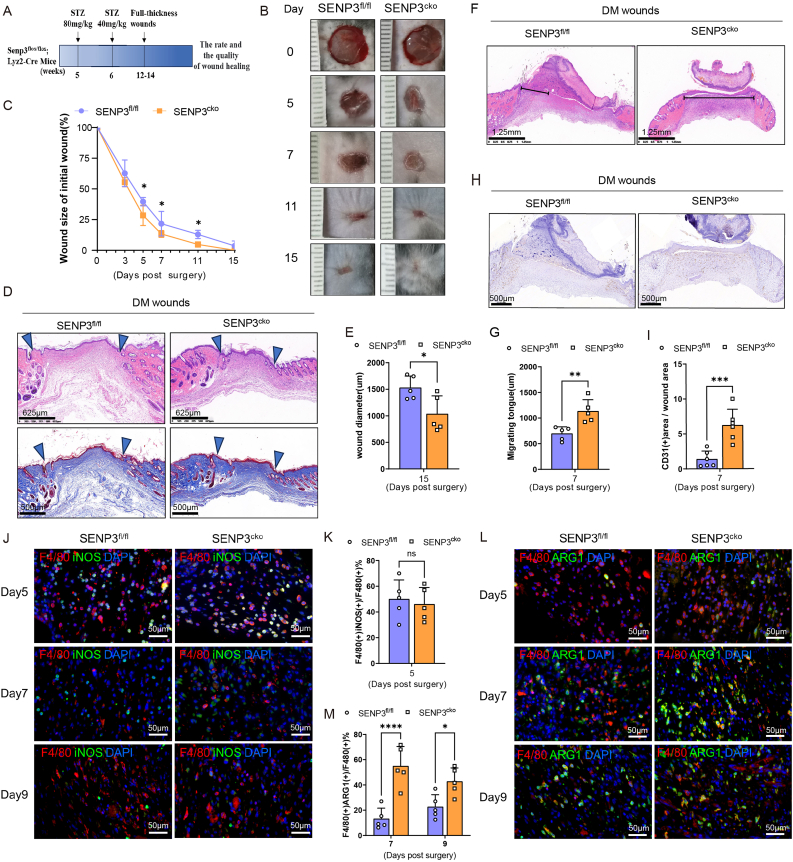
SENP3^cko^ mice exhibited an increased proportion of M2 macrophages and promoted diabetic wound healing. **(A)** SENP3^fl/fl^ (n = 19) and SENP3^cko^ (n = 19) mice were injected with STZ. After 6–8 weeks, two full-thickness wounds were created on each mouse. **(B–C)** Photographs of full-thickness wounds of diabetic SENP3^fl/fl^ and SENP3^cko^ mice were taken at the indicated time and representative photographs were shown (A); Statistical results of relative wound area (fold of the wound on Day 0) (n = 5) (B). **(D**–**E)** Skin wounds were collected at 15 days post-surgery and subjected to hematoxylin and eosin (HE) staining and Masson staining (D); statistical results of wound diameter measured in pictures from HE staining (n = 5) (E). **(F–I)** Skin wounds were collected at 7 days post-surgery and subjected to HE staining (F) and statistical results of migrating tongue on day 7 (n = 5) (G). Immunohistochemistry of CD31 (H) and statistical results of CD31 positive area/total area% (n = 6) (I). **(J**–**M)** Skin wounds were collected on days 5, 7, and 9. The skin wounds slices were subjected to immunofluorescence staining of F4/80 and iNOS (J) and statistics analysis of F4/80(+) iNOS (+)/F4/80(+) cells% in day 5 diabetic wounds from SENP3^fl/fl^ and SENP3^cko^ mice (K). Immunofluorescence staining of F4/80 and ARG1 (L) and statistical results of F4/80 (+) ARG1 cells/F4/80 (+) cells% of day 7 and day 9 diabetic wounds (M). Scale bars, as indicated in the figures. Data represents mean ± SD. The above results were acquired from four independent experiments. ***p < 0.001, **p < 0.01, *p < 0.05, unpaired *t*-test (C, E, G, I, K). ****p < 0.0001, *p < 0.05, two-way ANOVA (M).

Next, we conducted experiments on SENP3^fl/fl^ and SENP3^cko^ mice to investigate the impact of SENP3 expression on diabetic wound healing and macrophage polarization during the wound healing process. These mice were intraperitoneally injected with streptozotocin (STZ) to induce diabetes (Fig. 4A). The body weights and glucose levels of these mice were monitored regularly for up to 6 weeks. The results showed that the glucose levels in all mice were greater than 16.7 mmol/L, indicating successful induction of the diabetic model. The two groups of mice exhibited comparable body weights and blood glucose levels (Supplementary Figs. 3A and B). Co-staining of SENP3 and F4/80 in the wounds on day 5 confirmed that SENP3 was knocked out in macrophages (Supplementary Fig. 3C).

Full-thickness wounds were generated on these diabetic SENP3^fl/fl^ and SENP3^cko^ mice, and the speed and the quality of wound healing were accessed (Fig. 4A). Complete re-surfacing of the diabetic wounds occurred by day 15 in SENP3^cko^ mice, with visibly smaller wound areas than in the SENP3^fl/fl^ mice at days 5, 7, and 11 post-injury (Fig. 4B and C). HE and Masson staining on day 15 revealed that the wounds from the SENP3^cko^ mice had an increased number of dermal papillae, epidermal reticular ridges, well-constructed dermal and epidermal junctions, and a more compact dermal layer composed of collagens (Fig. 4D), with a decreased wound diameter, suggesting a greater healing speed (Fig. 4D and E). Moreover, the longer length of the migrating tongue on day 7 in wounds from SENP3^cko^ mice than in those from SENP3^fl/fl^ mice indicated faster re-epithelialization in SENP3^cko^ mice (Fig. 4F and G). Additionally, we evaluated vascularization in a wound by measuring the presence of CD31, a marker specific to endothelial cells. We found more CD31-positive vessels in the wounds of the SENP3^cko^ mice than in those of SENP3^fl/fl^ mice, suggesting more blood vessel formation in the wounds of the SENP3^cko^ mice (Fig. 4H and I). The above results suggest that SENP3 deficiency in macrophages significantly improved diabetic wound healing.

Diabetic wounds from SENP3^fl/fl^ and SENP3^cko^ mice were subjected to immunofluorescence assay to analyze macrophage polarization by co-staining F4/80 macrophage with iNOS as a marker of M1 and ARG1 as a marker of M2. As shown in Fig. 4J, F4/80+ iNOS + cells could be observed at day 5 post-injury but were barely observed at day 7 and day 9 in the wound tissues, with comparable proportions of F4/80+ iNOS + cells in the SENP3^fl/fl^ and SENP3^cko^ mice (Fig. 4K). Moreover, F4/80+ ARG1+ cells were observed at 7 and 9 days post-injury, but not at 5 days in the diabetic wounds from the SENP3^fl/fl^ and SENP3^cko^ mice (Fig. 4L). More importantly, the proportion of F4/80+ ARG1+ cells of wounds on days 7 and 9 in the SENP3^cko^ mice was greater than that of the SENP3^fl/fl^ group (Fig. 4M). These findings suggest that SENP3 deficiency in macrophages increases the proportion of M2 macrophages and promotes diabetic wound healing.

### 3.5 SENP3-driven ferroptosis is dependent on FSP1

**Fig. 5:**
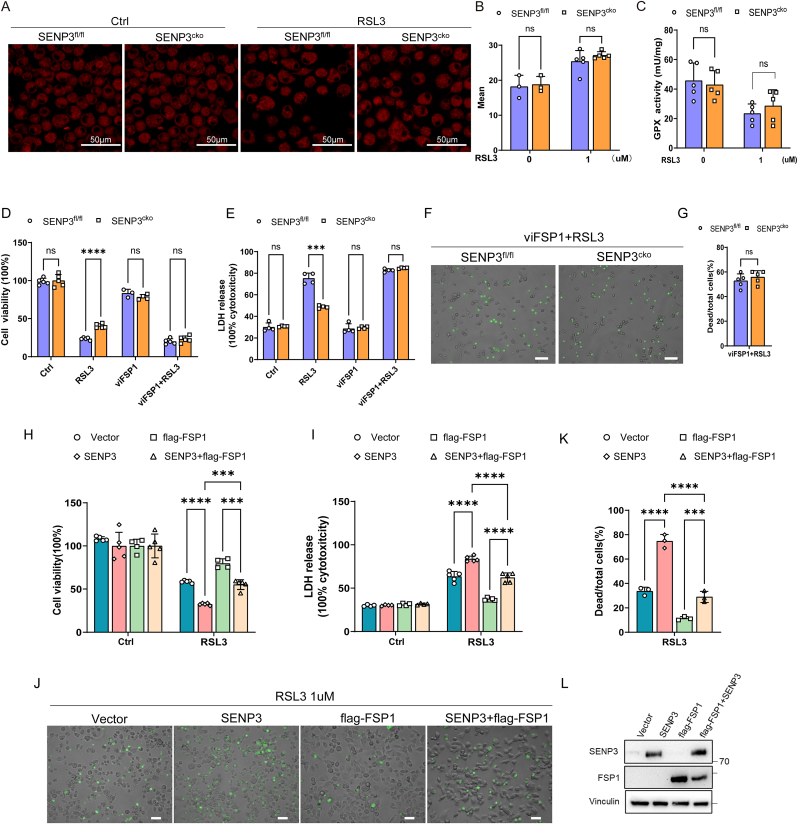
SENP3-driven ferroptosis is dependent on FSP1. **(A**–**B)** BMDMs from SENP3^fl/fl^ and SENP3^cko^ mice were treated with RSL3 (1 μM) for 4 h. FerroOrang staining of cells was completed (A). The statistics of mean fluorescence intensity were shown (B). **(C)** SENP3^fl/fl^ and SENP3^cko^ BMDMs were incubated with RSL3 for 2 h. GPX activity was evaluated. **(D**–**G)** SENP3^fl/fl^ and SENP3^cko^ BMDMs were pretreated with or without viFSP1 (1 μM) for 24 h followed by RSL3 (1 μM) incubation for 5 h. Next, the viability (D) and LDH release (E) of these cells were tested. In addition, SYTOX Green staining of these cells and statistics analysis of dead/total cells% were shown (F–G). **(H**–**L)** HT1080 cells were stable over expressed SENP3 or empty vector as control, with 3 × flag-FSP1 or 3 × flag-vector. These cells were subjected to RSL3 (1 μM) treatment for 5 h. Then, the viability (H) and LDH release (I) of vector, SENP3, flag-FSP1 and flag-FSP1+ SENP3 transfected HT1080 cells were tested. In addition, SYTOX Green staining of these cells (J) and statistics analysis of dead/total cells% (K) were shown. The expression of SENP3 or FSP1 of the four group cells was validated by IB (L). Scale bars, 100 μm or as indicated in the figures. Data are shown as means ± sd. Data are representative of two (A, B) or three independent experiments (D–L). ****p < 0.0001, ***p < 0.001, ns *p* > 0.05, multiple unpaired t-tests (B–E). ns *p* > 0.05, unpaired *t*-test (G). ****p < 0.0001, ***p < 0.001, two-way ANOVA (H, I, K).

The underlying mechanisms by which SENP3 regulates ferroptosis remain unclear. Ferroptosis is iron-dependent. Here, we used FerroOrang to test the accumulation of free iron (Fe^2+^). Increased Fe^2+^ levels were shown after BMDMs incubation with RSL3, but the Fe^2+^ levels in the SENP3^fl/fl^ and SENP3^cko^ BMDMs were comparable (Fig. 5A and B). These results indicate that the SENP3 sensitizing macrophages to RSL3-induced ferroptosis is independent of iron accumulation.

Considering that RSL3 affects GPX4 (glutathione peroxidase 4), GPX activity was assessed in the SENP3^fl/fl^ and SENP3^cko^ BMDMs. Both the SENP3^fl/fl^ and SENP3^cko^ BMDMs showed comparable decreases in GPX activity after RSL3 incubation (Fig. 5C). These results indicate that SENP3 deficiency did not affect GPX activity during RSL3-induced ferroptosis.

In addition to GPX4, FSP1 is important in preventing cells from ferroptosis [35]. To explore whether SENP3 sensitizing macrophages to RSL3-induced ferroptosis is FSP1-dependent, SENP3^fl/fl^ and SENP3^cko^ BMDMs were subjected to RSL3 with pretreatment of viFSP1 [36], an FSP1 inhibitor, for 24 h. Compared with SENP3^fl/fl^ BMDMs, SENP3^cko^ BMDMs showed an increase in cell viability, and a decrease in LDH release of the RSL3 group, while cell viability (Fig. 5D), LDH release (Fig. 5E), and percentage of dead cells (Fig. 5F and G) were comparable in the viFSP1+RSL3 group (Fig. 5D–G). These data suggest that the protective effect of SENP3 loss on ferroptosis was reversed by FSP1 inhibition, indicating that SENP3 may regulate ferroptosis through FSP1.

To confirm that SENP3 regulates ferroptosis through FSP1, we used HT1080, a widely used fibrosarcoma cell line that is commonly used in ferroptosis studies. Consistent with previous data, the overexpression of SENP3 in HT1080 cells sensitized them to ferroptosis induced by RSL3, while co-transfection with FSP1 inhibited the effects of SENP3 overexpression (Fig. 5H–K). We also verified the protein level of SENP3 and FSP1 expression in each group by IB (Fig. L). These results indicate that SENP3 sensitizes ferroptosis of macrophages is dependent on FSP1 and that SENP3 may affect the expression of FSP1.

### 3.6 SENP3 De-SUMOylates FSP1 at the K162 site

**Fig. 6:**
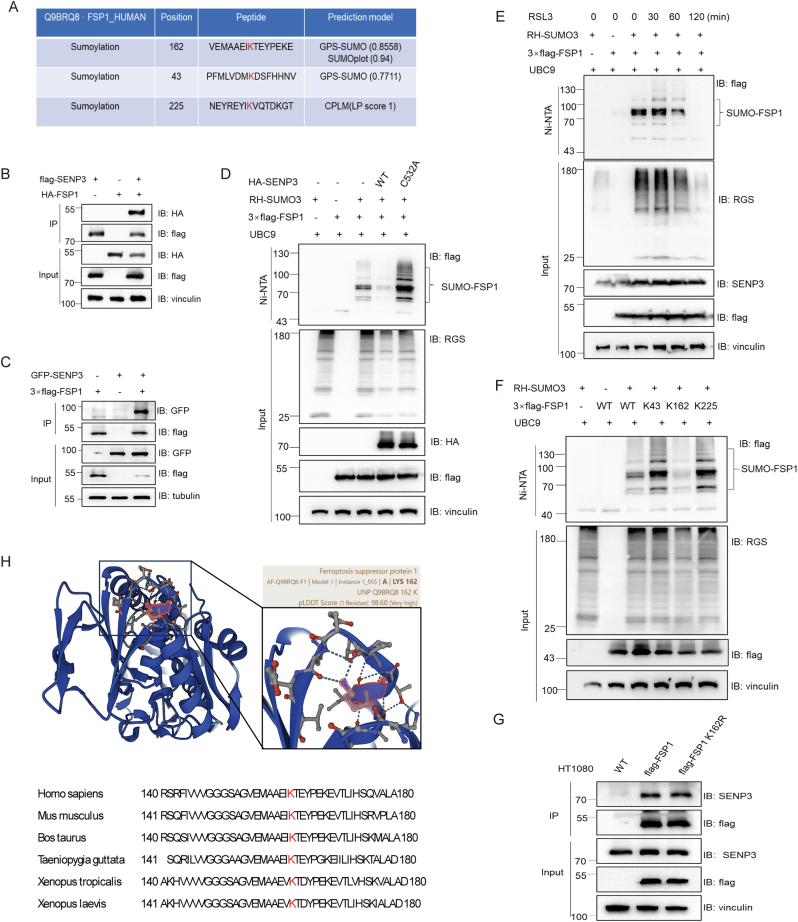
SENP3 De-SUMOylates FSP1 at the K162 site. **(A**) prediction of SUMO modification site of Q9BRQ8.FSP1_HUMAN using GPS-SUMO, SUMOplot, and CLPM. **(B–C)** HEK293T cells were co-transfected with co-transfected with flag–SENP3 and HA-FSP1 plasmids or GFP–SENP3 and 3 × flag-FSP1 plasmids with vectors as control for 36 h. Co-IP was performed using FLAG-M2 beads for immunoprecipitation and using anti-HA (B) or anti-GFP antibodies (C) for IB. **(D)** HEK293T cells were transfected with 3 × flag-FSP1, RH–SUMO3, UBC9, HA-SENP3, or HA-SENP3 C532A mutant with indicated vectors for 48 h. RH-SUMO3 was pulled down using Ni-NTA beads and then analyzed by IB as indicated. Close brace indicated SUMO3-conjugated FSP1. **(E)** HEK293T cells were transfected with 3 × flag-FSP1, RH–SUMO3, and UBC9 with indicated vectors for 48 h followed by stimulation with RSL3 (10 μM) for the indicated time. FSP1 that was conjugated with RH–SUMO3 was detected by Ni-NTA pull-down assay. **(F)** HEK293T cells were transfected with RH–SUMO3, UBC9, and 3 × flag-FSP1 or 3 × flag-FSP1 K43R, K162R, K225R mutants with indicated vectors for 48 h. Cells were lysed and RH–SUMO3 was pulled down using Ni-NTA beads and then analyzed by IB as indicated. Close brace indicated SUMO3-conjugated FSP1. **(G)** HT1080 cells were stably overexpressing 3 × flag-FSP1 or 3 × flag-FSP1 K162R. Co-IP was performed using FLAG-M2 beads for immunoprecipitation and using anti-SENP3 antibody for IB. **(H)** Structure of human FSP1 protein from AlphaFold Protein Structure Database. The K162 site in FSP1 is conserved. The sequences around FSP1 K162 from different species were aligned. Conserved lysine residues corresponding to human FSP1 are marked in red. WB data represents at least three independent experiments.

The enzyme SENP3, a SUMO-specific protease, is responsible for removing the SUMO from substrate proteins, affecting their stability, activity, and function [27,37]. We used SUMOylation prediction software to predict the possibility of SUMOylation of FSP1 (https://www.abcepta.com/sumoplot/; http://cplm.biocuckoo.cn/View.php?id=CPLM055952; https://sumo.biocuckoo.cn/userguide.php). The results showed that FSP1 has a high SUMO modification score according to all three kinds of SUMO prediction software (Fig. 6A).

Therefore, a HEK293T cell overexpression system was used to determine whether SENP3 interacts with FSP1. Co-IP assays were performed. The results indicated that SENP3 and FSP1 interact with each other (Fig. 6B and C). Next, we investigated whether FSP1 was subjected to SUMO3 modifications using HEK293T cells. SUMO3 conjugated to FSP1 exhibited bands at 100–130, 70–100, and 55–70 kDa, indicating poly-SUMO protein conjugation (Fig. 6D). Moreover, there was a marked increase in the SUMOylation of FSP1 with increasing RH–SUMO3 overexpression (Supplementary Fig. 4A). Wild-type (WT) SENP3 removed SUMO3 on FSP1 (Fig. 6D, Supplementary Fig. 4B), while SENP3 inactivating mutant (C532A) did not show such effects (Fig. 6D). To validate that the SUMO3 modification of FSP1 changed during ferroptosis, RH-SUMO and flag-FSP1 were overexpressed in HEK293T cells. After RSL3 induction, the SUMO3 conjugated FSP1 level dramatically decreased (Fig. 6E). These findings indicate that FSP1 is a SUMO3 substrate protein that can be de-SUMOylated by SENP3.

To identify the SUMOylation site of FSP1, plasmids were constructed for overexpression of flag-tagged WT-FSP1 or K43R–FSP1, K162R–FSP1, or K225-FSP1 according to the prediction software as shown in Fig. 6A. The results showed that the K162R mutation noticeably decreased SUMO3-conjugation with the FSP1 protein. However, the K43R and K225R mutations did not affect the SUMO bands (Fig. 6F). Moreover, HT1080 cells stably overexpressed WT-FSP1 or K162R–FSP1 followed by Co-IP assay. The results suggested that the FSP1 K162R mutant cannot affect the FSP1-SENP3 binding ability (Fig. 6G). These data confirmed that FSP1 is a substrate of SENP3 and that K162 is a dominant SUMO3 modification site of FSP1. The FSP1 structure suggests that K162 is conserved in species ranging from Xenopus to various mammals (Fig. 6H). These findings provide evidence that the site at K162 has the potential to serve as a regulatory site for FSP1 function.

Next, we tried to determine the possible mechanism by which SENP3 and SUMOylation regulate FSP1. From the results in Figs. 5L and 6C, and Supplementary Fig. 4B, the protein level of FSP1 decreased when SENP3 was co-transfected, indicating that the protein level of FSP1 may be affected by SENP3. Thus, we explored the effects of SENP3 and SUMOylation on the FSP1 protein level. The data suggested that the FSP1 protein level was increased in a dose-dependent manner by RH-SUMO3 overexpression (Supplementary Fig. 4C) and decreased by SENP3 overexpression (Supplementary Figs. 4D and E). Next, we explored whether SENP3 affects the FSP1 stability by overexpressing FSP1 in HT1080 cells with or without SENP3, and then these cells were subjected to the protein biosynthesis inhibitor cycloheximide. The results showed that the half-life of FSP1 was significantly shortened by SENP3 overexpression (Supplementary Fig. 4F). Moreover, NAC pretreatment increased the half-life of FSP1 by decreasing SENP3 accumulation (Supplementary Fig. 4G). Increased FSP1 level was also shown after stable knockdown of SENP3 in RAW264.7 cells and SENP3 knockout BMDMs at baseline and in response to RSL3 stimulation (Supplementary Figs. 4H and I). These data indicate that SENP3 reduces FSP1stability.

### 3.7 De-SUMOylation of FSP1 at the K162 site reversed its ability to inhibit ferroptosis

**Fig. 7:**
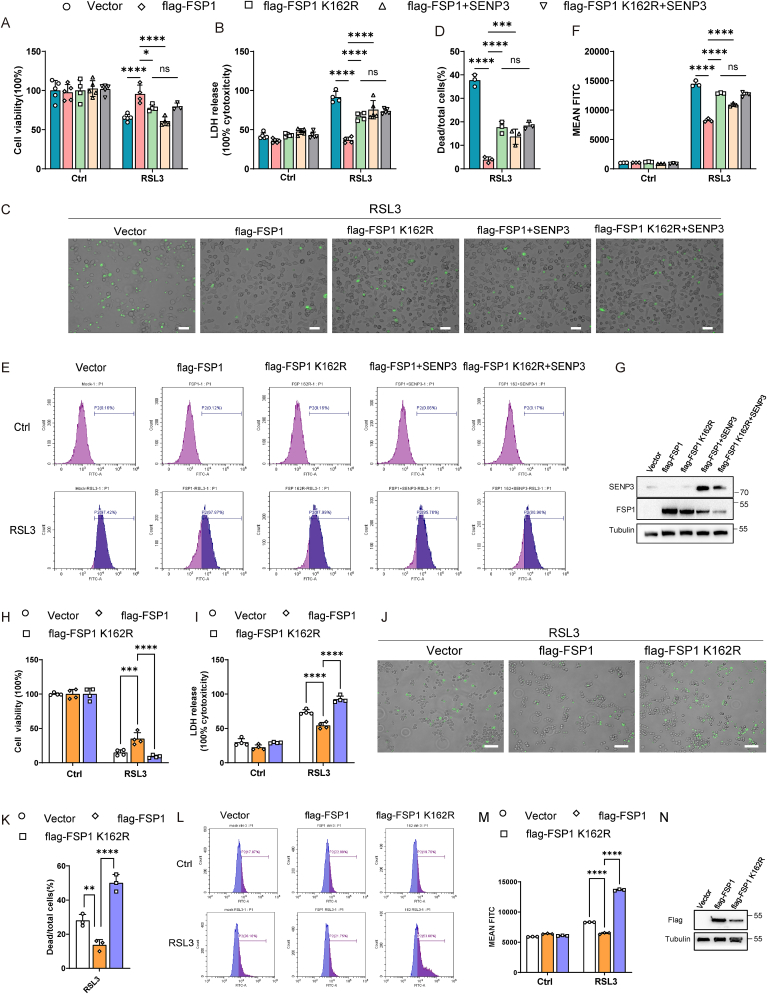
SENP3 de-SUMOylated FSP1 at the K162 Site and reversed its effect of inhibiting ferroptosis. **(A**–**D)** HT1080 cells were stably overexpressing 3 × flag-vector, 3 × flag-FSP1 or 3 × flag-FSP1 K162R, with SENP3 overexpression or empty vector, followed by RSL3 (1 μM, 5 h) incubation. Next, the viability (A) and LDH release (B) of cells were tested. SYTOX Green staining of cells (C) and statistics analysis of dead/total cells% were shown (D). **(E**–**G)** HT1080 cells were stably overexpressing 3 × flag-vector, 3 × flag-FSP1 or 3 × flag-FSP1 K162R, with SENP3 overexpression or empty vector, followed by RSL3 (1 μM, 1 h) incubation. Lipid peroxidation was evaluated by BODIPY 581/591 C11 staining(E). Statistics analysis of mean FITC intensity was shown (F). Western blots validated the expression of FSP1 and SENP3 expression in HT1080 cells which were transfected with 3 × flag-vector, 3 × flag-FSP1, or 3 × flag-FSP1 K162R, with SENP3 overexpression or empty vector (G). **(H–K)** RAW264.7 macrophages were stably overexpressing vector as control, 3 × flag-FSP1 or 3 × flag-FSP1 K162R followed by RSL3 (1 μM, 5 h) incubation. Next, the viability (H) and LDH release (I) of cells were tested. SYTOX Green staining of cells (J) and statistics analysis of dead/total cells% were shown (K). **(L**–**N)** RAW264.7 macrophages were stably overexpressing empty vector, 3 × flag-FSP1 or 3 × flag-FSP1 K162R followed by RSL3 (1 μM, 1 h) incubation. Lipid peroxidation was evaluated by BODIPY 581/591 C11 staining (L). Statistics analysis of mean FITC intensity was shown (M). Western blots validated the expression of FSP1 expression in RAW 264.7 cells which were transfected with vector, mouse flag-FSP1, or mouse flag-FSP1 K162R (N). Scale bars, 100 μm. Represents results of three independent experiments. ****p < 0.0001, ***p < 0.001, *p < 0.05, two-way ANOVA (A, B, F, H, I, M). ****p < 0.0001, ns *p* > 0.05, one-way ANOVA (D, K).

We next analyzed the regulatory effect of SUMOylation at the K162 site on FSP1 function. HT1080 cells were stably overexpressed flag-FSP1 or flag-FSP1 K162R with or without SENP3 overexpression. After RSL3 treatment, compared with control cells and flag-FSP1 K162R-overexpressing cells, FSP1-overexpressing cells had significantly greater cell viability and decreased LDH release (Fig. 7A and B). The SYTOX Green assay showed that there were fewer dead FSP1 overexpressing cells than control cells and FSP1 K162R overexpressing cells (Fig. 7C and D). Compared to control cells and flag-FSP1 K162R-overexpressing cells, flag-FSP1-overexpressing cells also exhibited decreased accumulation of hydroperoxy-lipids (Fig. 7E–G). These data indicate that mutating FSP1 at the K162 site inhibits the anti-ferropotic effect of FSP1. Moreover, simultaneous SENP3 overexpression significantly inhibited the anti-ferroptotic effect of FSP1 but did not exacerbate ferroptosis in FSP1 K162R-overexpressing cells (Fig. 7A–G). Collectively, these data suggested that the SUMO3 modification of FSP1 is vital for protection against ferroptosis.

To elucidate the protective effect of FSP1 and the K162 site mutant FSP1 on macrophage ferroptosis, mouse flag-FSP1, mouse flag-FSP1 K162R, or control plasmid was stably overexpressed in RAW 264.7 cells. Consistent with the results in HT1080 cells, compared with flag-FSP1 K162R-overexpressing cells, flag-FSP1-overexpressing RAW macrophages exhibited increased cell viability (Fig. 7H), reduced LDH release (Fig. 7I), fewer SYTOX Green-positive cells (Fig. 7J and K), and decreased accumulation of hydroperoxy-lipids (Fig. 7L and M). Overall, mutating FSP1 at the K162 site reversed its protective effect against RSL3-induced macrophage ferroptosis.

## 4 Discussion

It remains unknown whether SENP3 and SUMOylation regulate FSP1 and macrophage ferroptosis. In this study, we clarified that SENP3 can sensitize macrophages to RSL3-induced ferroptosis through de-SUMOylating FSP1 at the K162 site. We suggest that SENP3 inhibits the proportion of M2 macrophage by promoting ferroptosis to delay diabetic wound healing. These results will broaden the understanding of the biological roles of SENP3 and SUMOylation in regulating FSP1 and macrophage ferroptosis.

In the past decade, the regulatory mechanisms of protein post-translational modifications (PTMs) involved in ferroptosis have been gradually recognized for their importance. Several PTMs, including phosphorylation, ubiquitination, acetylation, and methylation, were suggested to be implicated in controlling ferroptosis [38]. However, much is still unknown about new PTMs involved in ferroptosis regulation [14,15]. Our study reports a novel role for SENP3 and SUMO3 modification in regulating the ferroptosis of macrophages. We observed that SENP3 quickly accumulated along with decreased SUMO3 modification during RSL3-induced macrophage ferroptosis. Moreover, SENP3 loss partly rescued macrophages from RSL3-induced ferroptosis. Our work highlights the function of SUMOylation and SENP3 in macrophage ferroptosis.

The dynamic balance of protein SUMOylation and de-SUMOylation, especially for SUMO2/3, is thought to be an important response for cells to oxidative stress [27,32]. Ferroptosis is driven by ROS accumulation due to excessive free iron and subsequent lipid peroxidation. These events are closely related to oxidative stress [39]. Improving antioxidant capacity or scavenging ROS can effectively enhance resistance to ferroptosis [40]. Our results suggested that SENP3, a protease specific to SUMO2/3 and sensitive to redox status, partly accounts for the facilitation of ferroptosis by ROS, which furthers our understanding of the mechanisms during ferroptosis.

GPX4 mediates the reduction of lipid peroxides [41]; FSP1 prevents lipid peroxidation by converting ubiquinone to ubiquinol [20,21]; and dihydroorotate dehydrogenase (DHODH) inhibits mitochondrial lipid peroxidation [42]. These proteins increase cell tolerance to ferroptosis. Although substrates closely related to ferroptosis, such as Keap1/Nrf2 [43], P53 [44], BECN1 [45], and NF2/YAP [46], can undergo SUMOylation, the role of SUMOylation in regulating key ferroptosis regulatory proteins is still unclear. Our results showed that FSP1 could be SUMOylated at the K162 site and de-SUMOylated by SENP3. We observed a decrease in the SUMOylation of FSP1 during RSL3-induced ferroptosis. De-SUMOylation of FSP1 at the K162 site reversed its ability to inhibit ferroptosis. These results revealed SUMOylation as a vital regulator of ferroptosis.

FSP1 suppresses ferroptosis by mediating ubiquinone (CoQ10) regeneration [20,21]. The catalytic activity of FSP1 requires the carboxy-terminal domain, which forms two active sites on either side of FAD and mediates functional dimerization. These sites function in reducing ubiquinone [47]. Myristoylation of FSP1, which can be inhibited by iFSP1, promotes its membrane localization, allowing it to exert its enzyme activity and inhibit ferroptosis [20,21]. Unlike iFSP1, icFSP1 initiates the relocalization and condensation of FSP1, thereby preventing ferroptosis induction. The FSP1 condensates induced by icFSP1 exhibit phase separation, a widely used mechanism to modulate biological activity [48]. Here, we found that SUMOylation of FSP1 is also important for its role in ferroptosis. The effect of FSP1 SUMOylation and SENP3 may be attributed to FSP1 SUMOylation on the stability of FSP1. However, we did not explore how SENP3 regulates the stability of FSP1, and whether the SUMOylation of FSP1 affects its subcellular localization, dimerization, or enzyme activity. Thus, the mechanism by which FSP1 is regulated upon SUMOylation needs to be further studied.

Our study suggests that the underlying mechanism by which SUMOylation and SENP3 affect ferroptosis is FSP1-dependent. The results will assist in the development of new approaches for treating multiple diseases, including chronic non-healing wounds. For example, the FSP1-dependent vitamin K metabolism was found to prevent ferroptosis [23]. It was reported that the topical use of CoQ10 [49], vitamin K [50], or NAC [51] in diabetic wounds can be beneficial via unidentified immunomodulatory mechanisms. Herein, combined with our discovery of increased SENP3 expression in diabetic wounds and that SENP3 inhibited the anti-ferroptosis effect of FSP1, the application of NAC to decrease SENP3 expression and as well as that of coenzyme Q10 or vitamin K to provide a substrate for FSP1 may have a synergistic effect on promoting wound healing. Further research in this area is warranted.

A pathologically increased M1/M2 proportion causes impaired tissue repair. Recently, Kagan and his colleagues showed that M1 macrophages are more resistant to ferroptosis than M2 macrophages, and the proportion of M2 macrophages can be increased by inhibiting ferroptosis in vivo [10]. Here, our results showed that the loss of SENP3 expression inhibited the sensitivity of M2 macrophages to ferroptosis. The pro-inflammatory effects of ferroptosis and the significance of M2 macrophages in tissue repair suggest that the regulation of ferroptosis by SENP3 is critical in managing inflammatory response in various degenerative conditions.

Previous studies have indicated that M2 macrophages have a relatively lower oxidative state than M1 macrophages [52]. We found that knockdown or knockout of SENP3 expression had no significant effect on the tolerance of M1 macrophages to ferroptosis but markedly inhibited ferroptosis in M2 macrophages. This may be due to the strong tolerance of M1 macrophages to ferroptosis, which is not easily induced in vitro, making the role of SENP3 unobservable. Conversely, this may be because M1 macrophages inherently possess a higher level of antioxidant systems, making the role of SENP3 in promoting ferroptosis less significant in these cells. In M1 macrophages, SENP3 is involved in regulating transcription factors and participating in inflammatory responses [24]. In contrast, M2 macrophages, in a relatively moderate redox state, have weaker antioxidant mechanisms and are more prone to ferroptosis. Therefore, the regulation of SENP3 on ferroptosis becomes apparent. This indirectly provides supportive evidence for the different redox states of macrophages in different polarization states.

Diabetic wounds are characterized by a failure to transition from an inflammatory phase to a regenerative phase, in which an elevated M1/M2 ratio plays a major pathological role. Previous studies have indicated that ROS contributes to impaired macrophage function and diabetic wound healing, but there is a lack of research on the direct removal of ROS. Our study describes the function of SENP3 and SUMO2/3 modification in macrophages in diabetic wounds, which was previously unknown. We found that SENP3 is highly expressed in diabetic wounds. Loss of SENP3 expression promoted diabetic wound healing and increased M2 macrophage infiltration. Our work suggests that protein SUMOylation is important for wound healing.

Iron overload [53] and increased ROS [54] have been identified as causes of unrestrained proinflammatory M1 macrophage population infiltration in chronic non-healing wounds. A recent study suggested that ferroptosis contributes to sustained inflammation and impaired diabetic wound healing [55]. However, the mechanisms and factors that determine ferroptosis in diabetic wounds are not fully understood. The high expression of SENP3 in diabetic wounds and its role in ferroptosis indicated that ferroptosis in diabetic wounds could be attributed to ROS-induced SENP3 accumulation. Inhibiting SENP3 or ferroptosis may be suitable for treating diabetic wounds.

## CRediT authorship contribution statement

**Xuelian Chen:** Writing – review & editing, Writing – original draft, Visualization, Supervision, Software, Project administration, Methodology, Investigation, Data curation. **Jizhuang Wang:** Writing – review & editing, Writing – original draft, Validation, Methodology, Investigation, Conceptualization. **Peilang Yang:** Project administration, Methodology, Conceptualization. **Hsin-Ying Liu:** Software. **Shan Zhong:** Methodology. **Chenghao Lu:** Methodology. **Min Gao:** Validation, Formal analysis. **Dan Liu:** Methodology. **Jie Zhang:** Funding acquisition. **Jiaqiang Wang:** Validation. **Shan Ma:** Methodology. **Wenao Wang:** Methodology, Investigation. **Hanting Zhu:** Methodology. **Xiong Zhang:** Writing – review & editing, Writing – original draft, Funding acquisition. **Yan Liu:** Writing – review & editing, Writing – original draft, Supervision, Project administration.

## Declaration of competing interest

The authors declare that they have no known competing financial interests or personal relationships that could have appeared to influence the work reported in this paper.

## Data Availability

Data will be made available on request.
