# Supplementary material for: SENP3 sensitizes macrophages to ferroptosis via de-SUMOylation of FSP1
Source: Redox Biol. 2024 Jul 14;75:103267. doi: 10.1016/j.redox.2024.103267 (PMC11301343; doi:10.1016/j.redox.2024.103267)
Supplement: Multimedia component 1 [file mmc1.docx]

**Supplementary data:**

**Table 1 The primers and shRNA sequences used in this study**

| Name of primer | Sequence |
| --- | --- |
| **For mutant constructs** |  |
| human FSP1-K43-R | cattgtggtggaaggagtccCtcatgtccaccagcatgaag |
| human FSP1-K43R-F | cttcatgctggtggacatgaGggactccttccaccacaatg |
| human FSP1-K162R-F | ggagatggcagcagagattaGaacagaatatcctgagaaag |
| human FSP1-K162R-R | ctttctcaggatattctgttCtaatctctgctgccatctcc |
| human FSP1-K225R-F | gagtatcgagagtacatcaGagtgcagacggacaaaggcac |
| human FSP1-K225R-R | gtgcctttgtccgtctgcactCtgatgtactctcgatactc |
| mouse FSP1-K162R-F | gcagcagagattaGaaccgagtaccctg |
| mouse FSP1 K162R-R | tcggttCtaatctctgctgccatctct |
| **Oligonucleotide sequence of shRNA** | |
| shRNA-SENP3 | ggatgctgctctactcaaa |

**Table 2 The plasmids used in this study**

| Plasmids | Scoure |
| --- | --- |
| RH-SUMO3 | (Lao, Yang et al. 2018) |
| UBC9 | (Lao, Yang et al. 2018) |
| HA-SENP3 | (Zhang, Yang et al. 2020) |
| HA-SENP3 C532A mutant | (Zhang, Yang, et al. 2020) |
| GFP-SENP3 | (Liu, Guo et al. 2020) |
| flag-SENP3 | (Lao, Yang et al. 2018) |
| SENP3 | Shanghai Xitu Biotechnology Co., Ltd |
| 3×flag-hFSP1 | Shanghai Xitu Biotechnology Co., Ltd |
| 3×flag-hFSP1 K43R | This paper |
| 3×flag-hFSP1 K162R | This paper |
| 3×flag-hFSP1 K225R | This paper |
| 3×flag-mFSP1 | Shanghai Xitu Biotechnology Co., Ltd |
| 3×flag-mFSP1 K162R | Shanghai Xitu Biotechnology Co., Ltd |
| HA-FSP1 | This paper |
| 3×flag-pLVML-CMV-IRES-puro | Shanghai Xitu Biotechnology Co., Ltd |
| pLVX-EF1a-IRES-puro | Shanghai Xitu Biotechnology Co., Ltd |

**Figure 1. The accumulation of SENP3 decreased SUMO2/3 Modification during RSL3-induced ferroptosis of macrophages.**

(A-B) RAW 264.7 macrophages were stimulated with RSL3 (1 μM) for the indicated time, and SUMO-1 modification was measured by IB (A); the relative levels of SENP3, SUMO2/3, and SUMO1 were normalized to actin (B).

(C-D) RAW 264.7 macrophages stable knockdown of SENP3 or not were constructed by sh-SENP3 and sh-NC. These cells were then incubated with RSL3 (1 μM) for the indicated time, and SENP3 and SUMO2/3 modification were monitored by IB (C); the relative level of SUMO2/3 was normalized to tubulin (D).

(E-F) RAW 264.7 cells were stimulated with RSL3(1 μM) in the presence or absence of 5 mM NAC for the indicated time. NAC was pretreated for 4 hours. SENP3 accumulation was monitored by IB (E); the relative level of SENP3 normalized to tubulin (F).


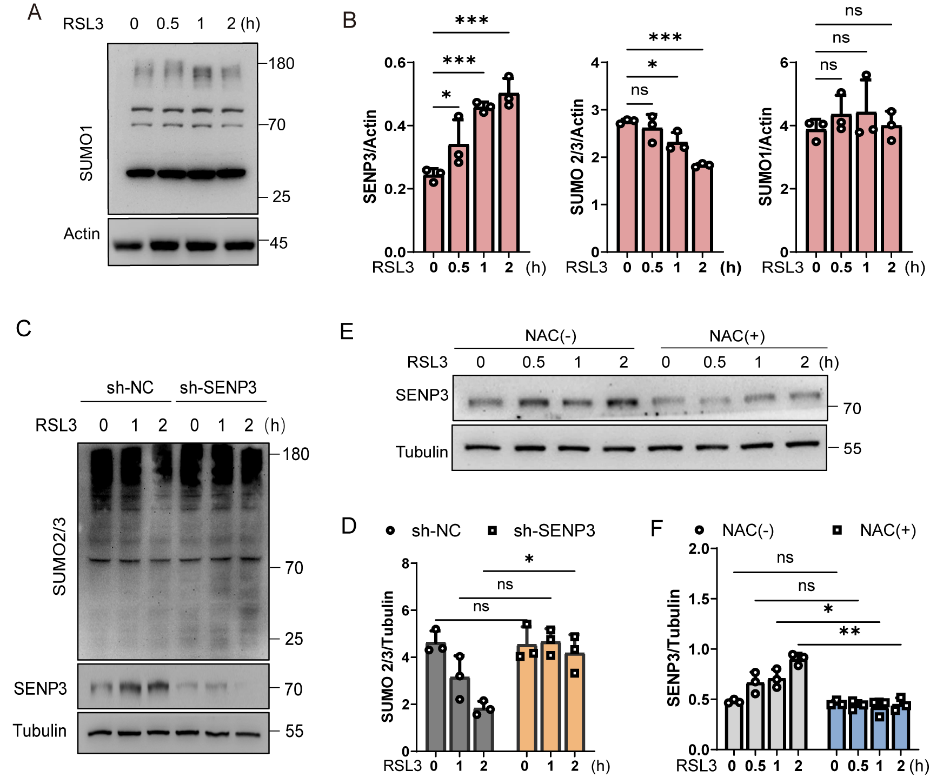


**Figure 2. Dynamic changes of SENP3 expression during normal wound healing process.** Full-thickness wounds were created on C57BL/6 wild-type mice and collected at the indicated time. immunohistochemistry of SENP3. Scale bars, 50 μm.

**
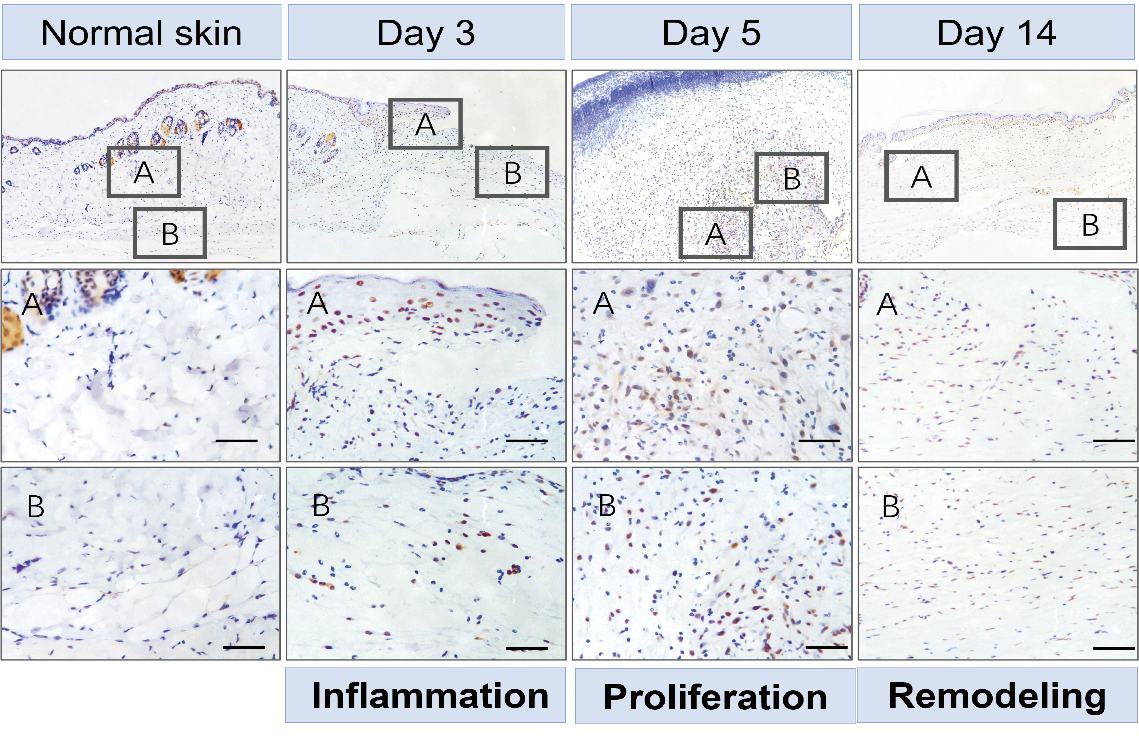
**

**Figure 3 Body weight and blood glucose levels between the SENP3^fl/fl^ and SENP3^cko^ mice after STZ injection are comparable**. After STZ injection, body weight and blood glucose levels were monitored every week of SENP3^fl/fl^ and SENP3^cko^ mice (A, B)**.** Co-staining of SENP3 and F4/80 in the day 5 diabetic wounds confirmed that SENP3 was knocked out in macrophages (C). Scale bars, 50 μm.

**
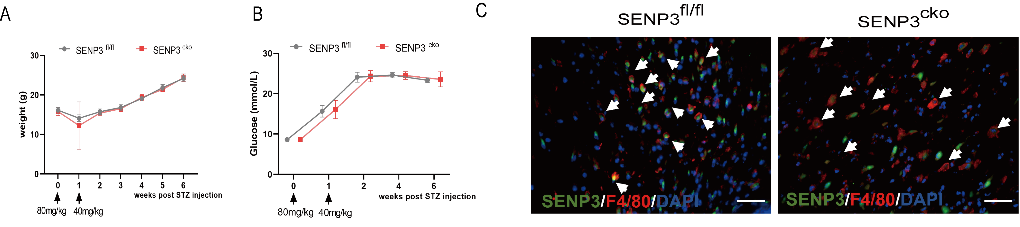
**

**Figure 4** **SENP3 De-SUMOylates FSP1 and inhibited its stability.**

**(A)** HEK293T cells were transfected with 3×flag-FSP1, UBC9, and an increasing dose of RH–SUMO3 (two or three times of flag-FSP1) which was two or three for 48 hours. RH-SUMO3 was pulled down using Ni-NTA beads and then analyzed by IB as indicated. Close brace indicated SUMO3-conjugated FSP1.

**(B)** HEK293T cells were transfected with 3×flag-FSP1, RH–SUMO3, UBC9 and GFP--SENP3 for 48 hours. RH-SUMO3 was pulled down using Ni-NTA beads and then analyzed by IB as indicated. Close brace indicated SUMO3-conjugated FSP1.

**(C)** HEK293T cells were transfected with 3×flag-FSP1, and an increasing dose of RH–SUMO3 for 36 hours. The expression of FSP1 was monitored by IB.

**(D, E)** HEK293T cells were transfected with 3×flag-FSP1 and an increasing dose of HA-SENP3 or GFP-SENP3 for 36 hours. The expression of FSP1 was monitored by IB.

**(F)** HT1080 cells stably overexpressing 3×flag-FSP1 with or without SENP3 were subjected to protein synthesis inhibitor cycloheximide (CHX, 200 ug/ml) for the indicated time. Lysates were prepared and the protein level of FSP1 was analyzed by IB.

**(G)** HT1080 cells stably overexpressing 3×flag-FSP1 were pretreated with or without NAC (5 mM) and subjected to CHX (200 ug/ml) for the indicated time. Lysates were prepared and the protein level of FSP1 was analyzed by IB. 3

**(H)** RAW 264.7 macrophages stable knockdown of SENP3 or no t were constructed by sh-SENP3 and sh-NC. These cells were then incubated with RSL3 (1 μM) for 2 hours, and SENP3 and FSP1 were monitored by IB.

**(I)** BMDMs from SENP3^fl/fl^ and SENP3^cko^ mice were incubated with IL-4 (25 ng/ml; M2 macrophages) for 24 hours. These cells were then incubated with RSL3 (1 μM) for 2 hours, and SENP3 and FSP1 were monitored by IB.

**
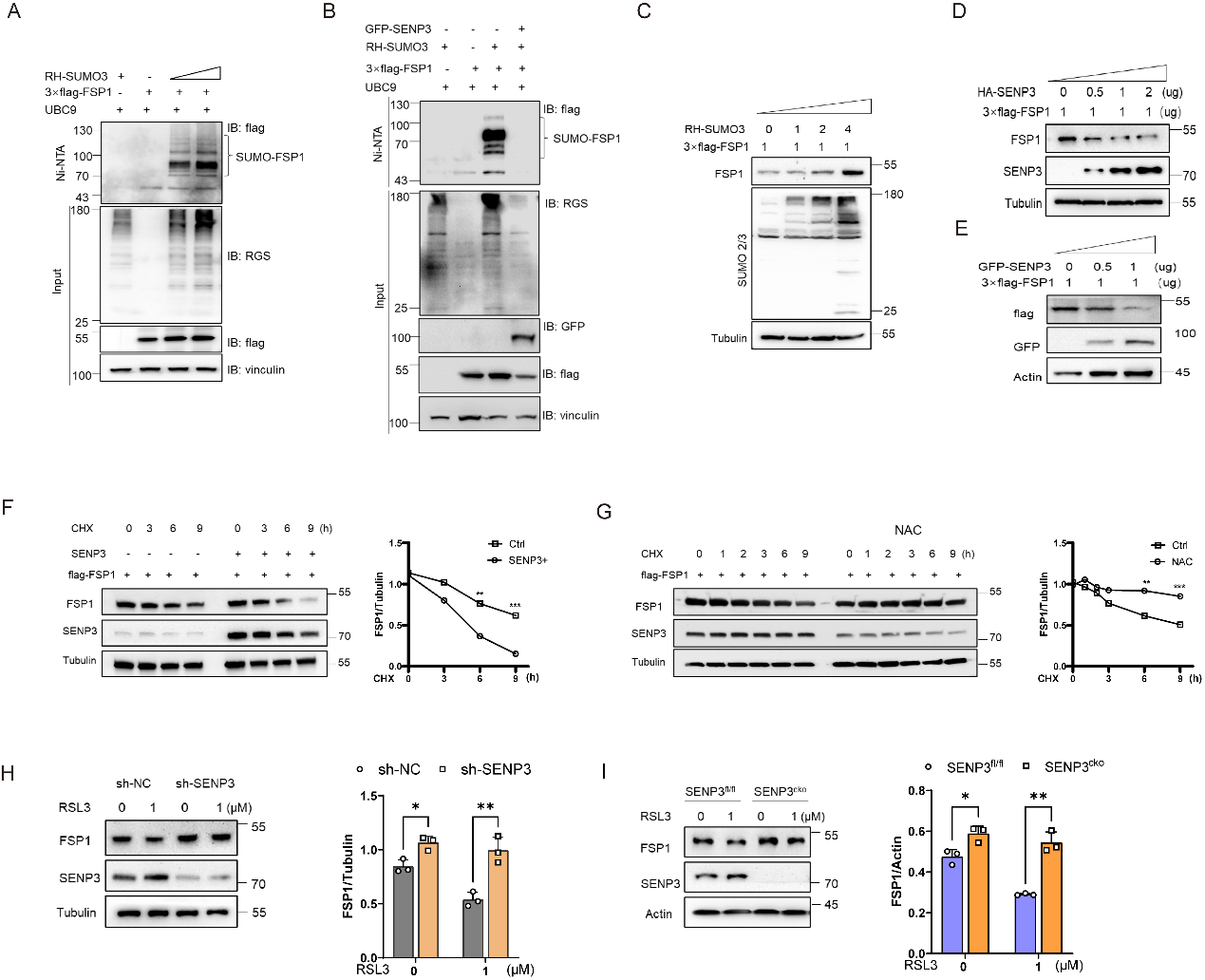
**
